# Post-laparotomy Hypoxia: A Case Series

**DOI:** 10.7759/cureus.28096

**Published:** 2022-08-17

**Authors:** Pradeep C Sharma, Neha Mahajan, Nidhi Uniyal, Rehnuma Ansari, Yashendra Sethi

**Affiliations:** 1 Department of Surgery, Government Doon Medical College, Dehradun, IND; 2 Department of Medicine, Government Doon Medical College, Dehradun, IND

**Keywords:** post-laparatomy hypoxia, postoperative hypoxia, postoperative care, abdominal surgery, explorative laparotomy, post operative hypoxia

## Abstract

Postoperative hypoxia is a challenge for surgeons. With the advent of better anesthesia and minimal access surgical techniques, the incidence of postoperative hypoxia in elective cases has decreased. However, the incidence in life-saving emergency procedures still poses a possible threat, and cases seem under-reported. We report a series of five cases of postoperative hypoxia after laparotomy. These cases comprise mesenteric laceration, proximal jejunal perforation, perforated duodenal ulcer, abdominal tuberculosis, and fall from height. Despite different etiologies, they landed up with the complication of postoperative hypoxia, which was attributable to the type of procedure they underwent and not the indication of the procedure itself. Thus, they form an interesting collection of post-laparotomy hypoxia cases. We present them with a compilation of probable causes of postoperative hypoxia in such cases.

Postoperative hypoxia presents a diagnostic challenge and requires timely suspicion, prompt intervention to eliminate the cause, and good postoperative care. The major causes include incomplete lung re-expansion, pain-induced restriction in chest-wall/diaphragm mobility, prolonged surgery, a complication of pre-existing lung disease, residual effects of some drugs, and iatrogenic causes. We, therefore, recommend the use of postoperative oxygen support and diligent monitoring of vitals in all cases of laparotomy, allowing prompt and timely patient management. Future studies are warranted to explore the prevalence and possible causes of post-laparotomy hypoxia.

## Introduction

Postoperative pulmonary complications (PPCs) are associated with about 20% mortality and are usually life-threatening. The International Classification of Diseases (ICD-11) has described postoperative/postprocedural respiratory failure as the need for ventilation for more than 48 hours after surgery or re-intubation with mechanical ventilation post-extubation. The cases for the same are evolving and are influenced by both the type of surgery and the length of hospital stay [1-3]

The most common threat is postoperative hypoxemia. It is one of the most dangerous complications, which increases postoperative morbidity [4]. The early postoperative period is a 'high-risk' time for the occurrence of hypoxemia [5]. Postoperative hypoxemia may occur secondary to the problem of gas exchange during anesthesia, which may continue in the early postoperative period [6]. It is highly evident that the physiological response of the operated patient is not reversed immediately after arriving in the recovery room and the patient stays at high risk of complications [7].

Hypoxemia can be seen with any type of procedure or anesthesia [4]. Among postoperative complications, PPCs dominate mortality and morbidity. It has been revealed that postoperative hypoxemia affects 30-50% of cases after abdominal surgery [8]. Postoperative hypoxia is expected to be more common in longer surgeries. The literature on postoperative hypoxia after laparotomy is very limited. We report a case series of post-laparotomy hypoxia and follow this with a discussion to add to the literature on the causes of these complications and possible interventions to avoid them.

## Case presentation

Case 1

**Figure 1:**
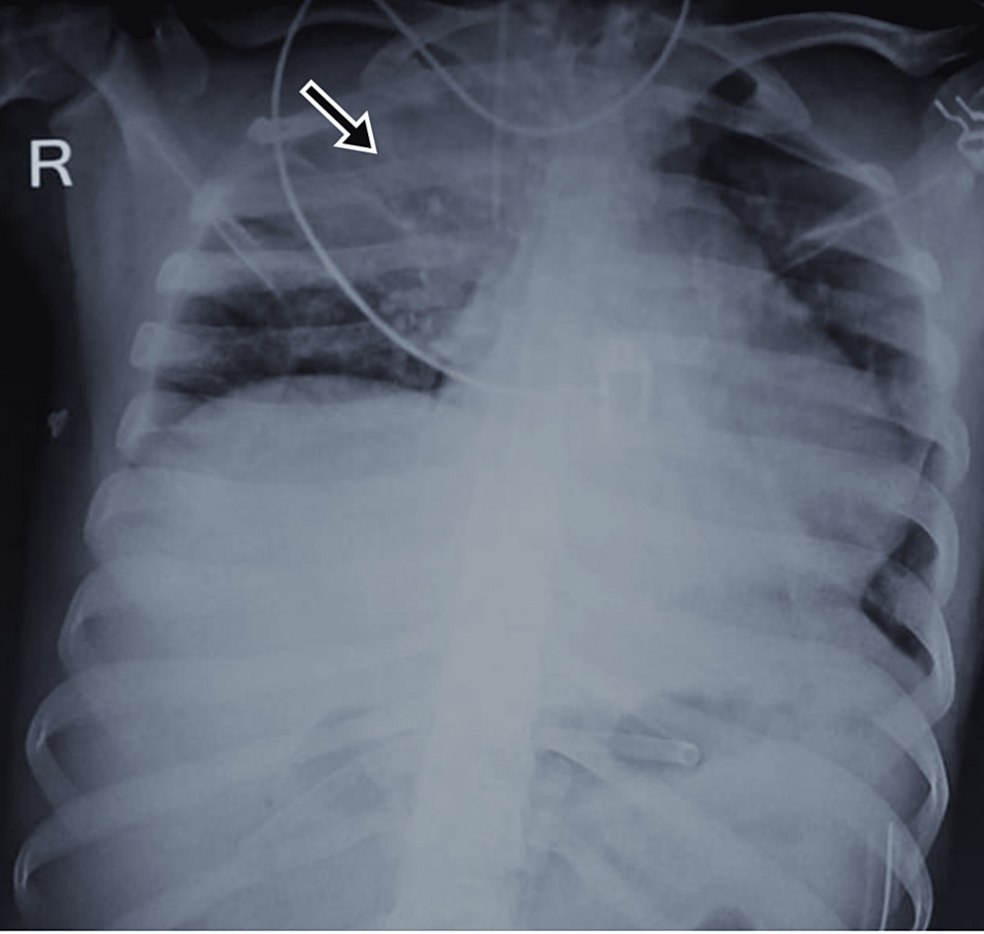
CXR of Case 1 showing right upper lobe consolidation (black arrow) CXR: chest x-ray

A 23-year-old male with an alleged history of road traffic accident reported to the ED with abrasions on his lower limbs and severe abdominal pain. On examination, the vitals were stable and the abdomen was rigid and had diffuse tenderness. USG imaging revealed free peritoneal fluid and blood investigations reported hemoglobin of 7.2 g/dL. He was a suspected case of hemoperitoneum and was immediately taken for exploratory laparotomy. Intraoperative findings of mesenteric laceration and perforation at the proximal jejunum were noted. He was operated on for mesenteric repair, segmental intestinal resection, and primary anastomosis. The patient was responding well after surgery and postoperative blood transfusions but the peripheral capillary oxygen saturation (SpO2) suddenly began to fall on day five after the surgery. The chest x-ray revealed the right upper lobe consolidation suggestive of pneumonitis (Figure 1). The patient had no history of respiratory illness or smoking. The patient was diagnosed as a case of sepsis and acute respiratory distress syndrome (ARDS) and was treated with meropenem, vancomycin, and gentamycin as per culture sensitivity. The patient initially recovered but then deteriorated and could not be saved.

Case 2

**Figure 2:**
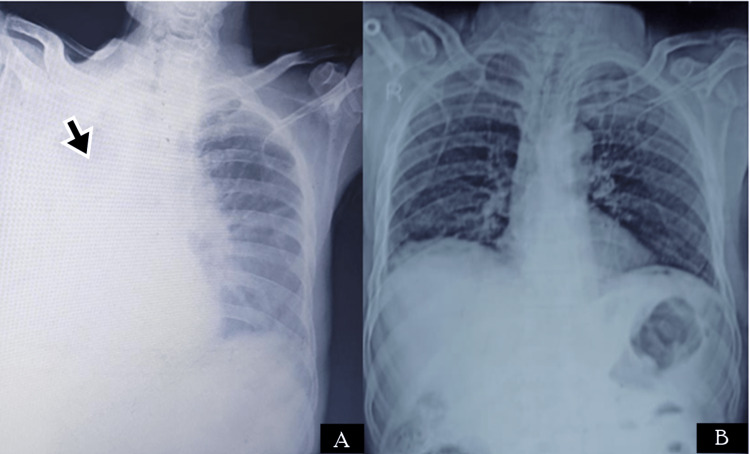
Radiological images of Case 2. (A) CXR showing right lung homogenous opacity (black arrow) suggestive of resorption collapse due to mucus plug; (B) CXR after the removal of the mucous plug CXR: chest x-ray

A 55-year-old male presented to the ED with complaints of upper abdominal pain and palpitations. On examination, he had tachycardia (heart rate: 123 bpm), and other vitals were within normal limits. The abdomen was rigid and had diffuse tenderness. The patient gave a history of previous treatment for duodenal ulcers, for which he took irregular treatment. Imaging reported signs of perforation and the patient was immediately taken for laparotomy. The patient was operated on; he underwent Graham omental patch and a thorough abdominal lavage was done. The patient was recovering well in the postoperative period when on day four after the surgery, he developed a sudden fall in oxygen saturation. He was a chronic smoker and was a known case of chronic obstructive pulmonary disease (COPD). CXR revealed a homogenous opacity on the right side of the chest (Figure 2A) and bronchoscopy revealed a mucus plug in the right bronchus. The mucus plug was removed and he was treated with broad-spectrum antibiotics and was advised to do incentive spirometry. Postoperative chest physiotherapy was also done. The patient responded well, he could maintain normal oxygen saturation within seven days of treatment, and also showed recovery on CXR (Figure 2B). The patient did not report any complications in follow-up.

Case 3

**Figure 3:**
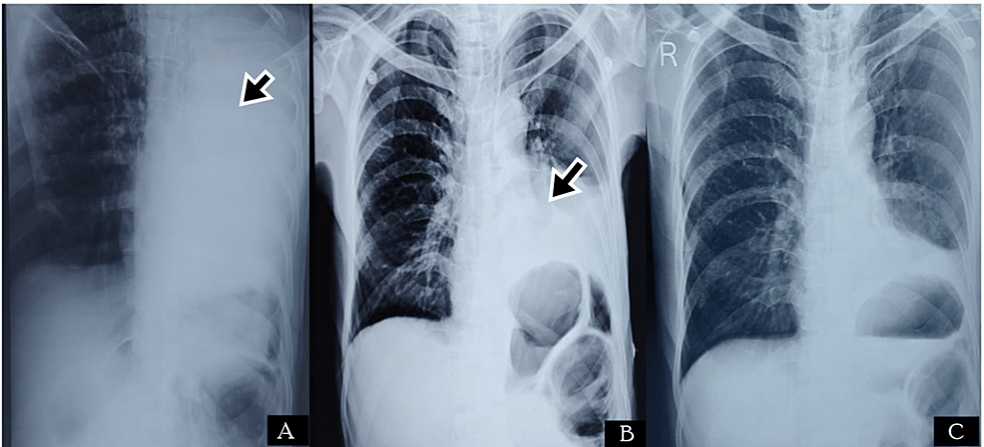
Radiological images of Case 3. (A) CXR showing complete homogenous opacification of the left lung (black arrow); (B) CXR after four days of treatment showing left lower zone homogenous opacity (black arrow) with left CP angle obliteration, suggestive of left lung basal lobe atelectasis with pleural effusion; (C) CXR after two weeks of recovery CXR: chest x-ray; CP: costophrenic

**Figure 4:**
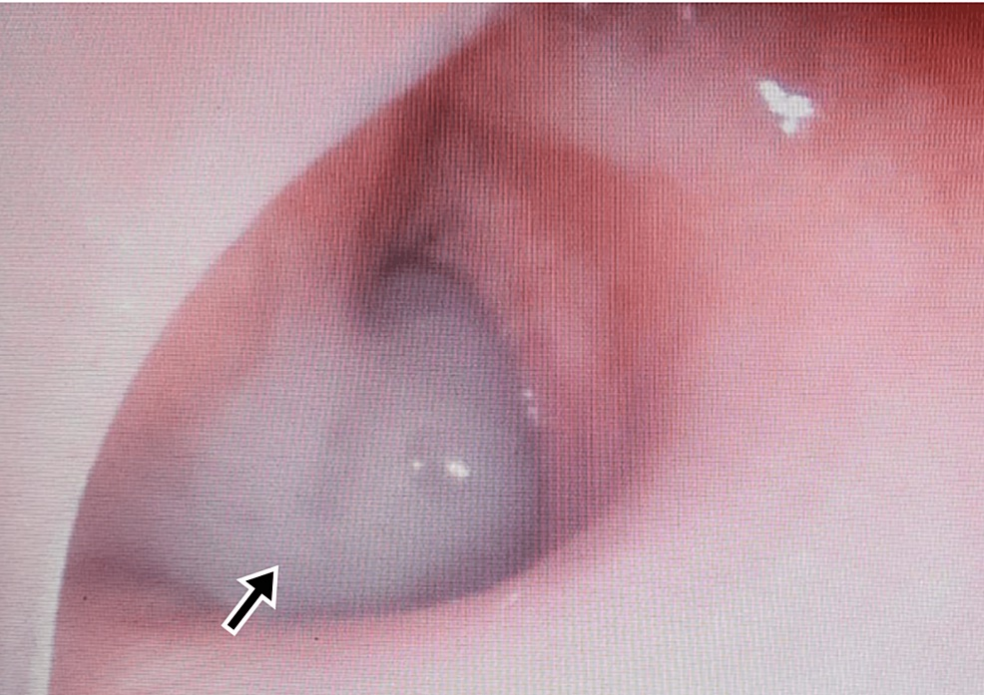
Bronchoscopy of Case 3 showing mucus plug

A 65-year-old male presented to the ED with complaints of severe upper abdominal pain. On examination, he had tachycardia (HR - 109 bpm), and other vitals were within normal limits. The abdomen was rigid and tenderness was present in the epigastric region. He had a previous history of burning sensation in the epigastric region, which was relieved on taking food. Imaging reported signs of perforation, and the patient was immediately taken for laparotomy. He was operated on with a pedicled omental flap and a thorough abdominal lavage was done. He was recovering well in the postoperative period when on day two after the surgery he developed a sudden fall in saturation. The CXR revealed homogenous opacity on the left side (Figure 3) and bronchoscopy revealed a mucus plug in the left bronchus (Figure 4). He was treated with broad-spectrum antibiotics and was advised to do incentive spirometry. Postoperative chest physiotherapy was also done. He responded well and could maintain normal oxygen saturation within four days of treatment. He did not report any complications in follow-up.

Case 4

**Figure 5:**
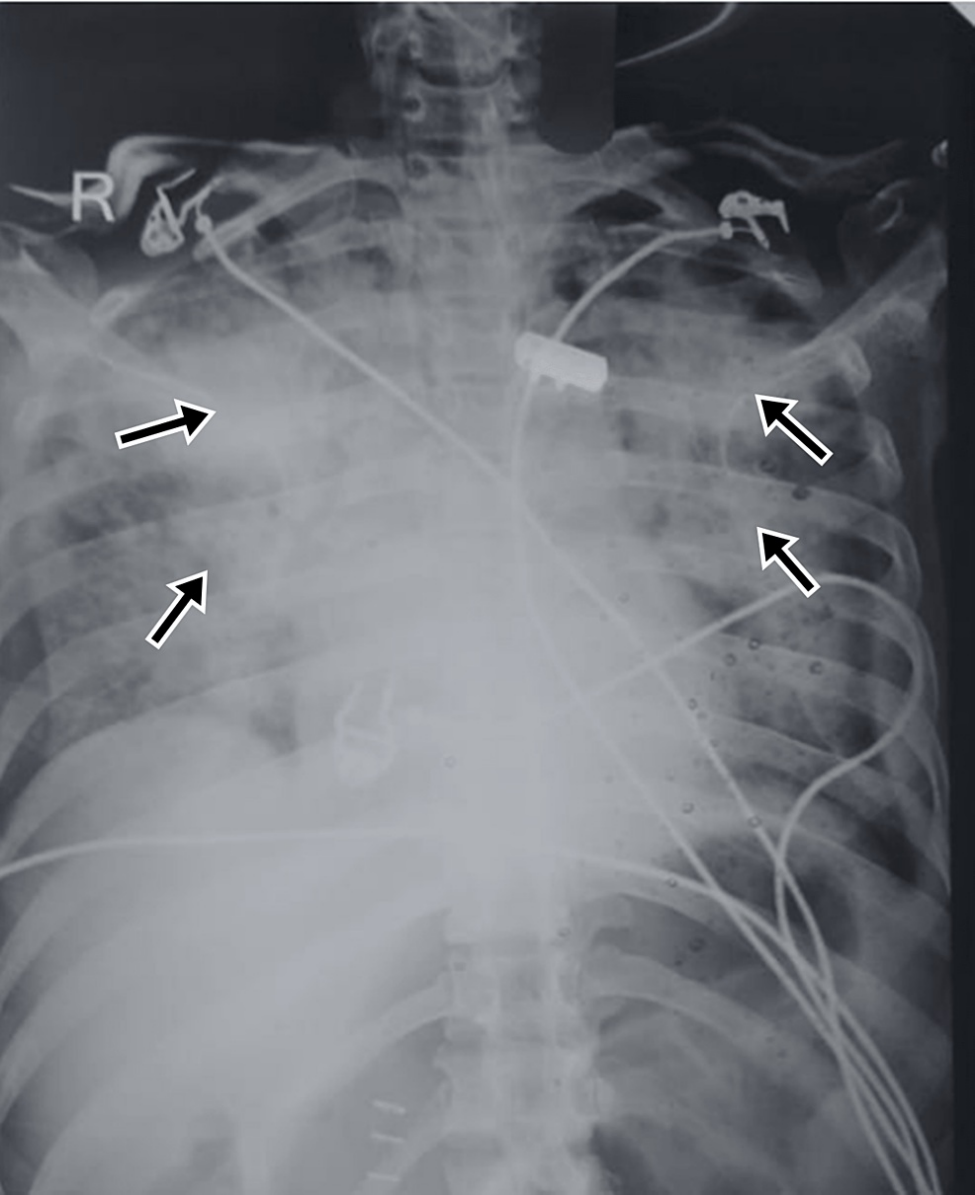
CXR of Case 4 showing bilateral non-homogenous opacities (black arrows) CXR: chest x-ray

A 50-year-old HIV-positive male with a history of pulmonary tuberculosis five years back reported to the ED with complaints of severe upper abdominal pain. On examination, he had stable vitals, but a per-abdominal exam revealed epigastric tenderness, abdominal rigidity, and rebound tenderness. The patient was investigated and imaging studies were done, which revealed free peritoneal fluid, and the patient was planned for laparotomy. A thorough abdominal lavage was done and a defect of 1.5cm was repaired in the proximal part of the duodenum. The patient developed the complication of fecal fistula and was treated with broad-spectrum antibiotics. The patient was recovering well but developed a sudden fall of saturation on day three after the surgery. His previous CXRs had perihilar pulmonary infiltrates but the recent one developed non-homogenous opacities bilaterally (Figure 5), which were diagnosed as *Pneumocystis carinii* infection on the basis of sputum microscopy. The patient was treated with broad-spectrum antibiotics, sulfamethoxazole+trimethoprim. The patient was advised to do incentive spirometry and postoperative chest physiotherapy was done. He responded well to treatment and recovered largely within two weeks. No respiratory complications were reported in follow-up.

Case 5

**Figure 6:**
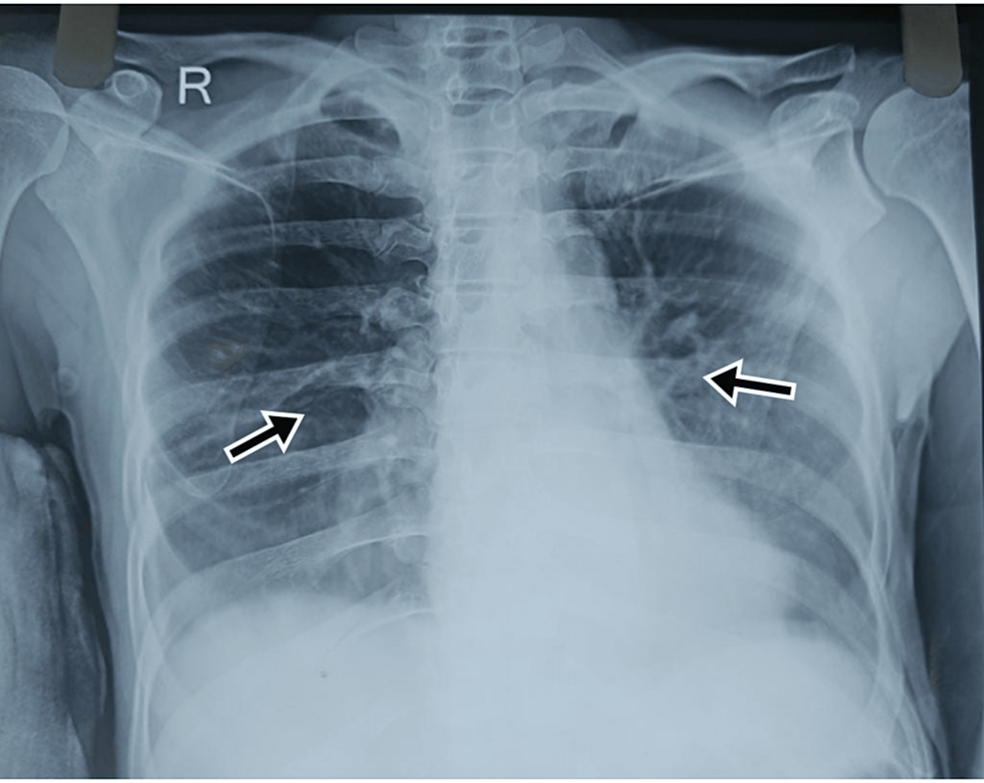
CXR revealed haziness on both sides of the chest suggestive of bilateral lower zone pneumonitis (black arrows) CXR: chest x-ray

A 49-year-old male was reported to the ED with an alleged history of fall from height. On examination, the patient had a feeble pulse and tachycardia. The abdomen was soft but had tenderness in the right hypochondriac region, epigastric region, and left hypochondriac region. He was referred to the medical college from a peripheral hospital and his investigations revealed a right intertrochanteric fracture and hemoglobin of 7.2 g/dL. Further investigations of USG raised suspicion of splenic injury. A non-contrast computerized tomography (NCCT) abdomen revealed a grade IV splenic injury and minor liver laceration. The patient was taken for emergency laparotomy and he underwent splenectomy with a repair of liver laceration. Adequate blood transfusions and fluid replacements were done. The patient developed postoperative hypoxia on day two of surgery; a CXR revealed haziness on both sides of the chest suggestive of bilateral lower zone pneumonitis (Figure 6). The patient was treated with antibiotics, incentive spirometry, and chest physiotherapy. He recovered well and was later operated on for the intertrochanteric (IT) fracture. The patient was vaccinated for capsulated organisms and counseled for post-splenectomy care. He recovered well and had no further complications to report in follow-up.

The comparative details of all the five cases reported under this series are given in Table 1.

**Table 1:** Comparison of reported cases of postoperative hypoxia after laparotomy WNL: within normal limits; COPD: chronic obstructive pulmonary disease; ABG: arterial blood gas; CXR: chest x-ray

Serial number	Age/sex	Co-morbidities	Past history of respiratory illness	Previous CXR	History of smoking	General Anesthesia	Duration of surgery (hours)	Pre-anesthetic medications used	Post-op hypoxia on day	Probable cause of Hypoxia	ABG
1	23/M	None	-	WNL	-	Isoflurane	2 hrs 30 min	Ondansetron, glycopyrrolate, fentanyl, midazolam	5	Sepsis secondary to pneumonia; Pain-induced restriction of diaphragmatic mobility	pH: 7.20 PaO2: 79mmHg PaCO2: 30 mmHg HCO3: 27 mEq/L S. Lactate: 3 mmol/L
2	55/M	COPD	COPD	Hyperinflated lungs	Chronic smoker - 80 pack years	Isoflurane	3 hrs	Ondansetron, glycopyrrolate, fentanyl, midazolam	4	Mucus plug obstruction of bronchus; Pain-induced restriction of diaphragmatic mobility	pH: 7.43 PaO2: 82mmHg PaCO2: 43mmHg HCO3: 25 mEq/L
3	65/M	Hypertensive	-	WNL	-	Isoflurane	2 hrs	Ondansetron, glycopyrrolate, fentanyl, midazolam	2	Secondary left lung lower zone pneumonia with left bronchus mucus plug obstruction	pH: 7.38 PaO2: 86mmHg PaCO2: 38mmHg HCO3: 26 mEq/L
4	50/M	HIV	*Pneumocystis carinii* infection	Perihilar pulmonary infiltrates	-	Isoflurane	3 hrs	Ondansetron, glycopyrrolate, fentanyl, midazolam	3	Flaring up of *Pneumocystis carinii* infection	pH: 7.35 PaO2: 77 mmHg PaCO2: 39 mmHg HCO3: 24 mEq/L
5	49/M	None	-	WNL	-	Isoflurane	2 hrs	Ondansetron, glycopyrrolate, fentanyl, midazolam	2	Pain-induced restriction of Diaphragmatic mobility	pH: 7.43 PaO2: 89 mmHg PaCO2:35 mmHg HCO3: 23mEq/L

## Discussion

**Figure 7:**
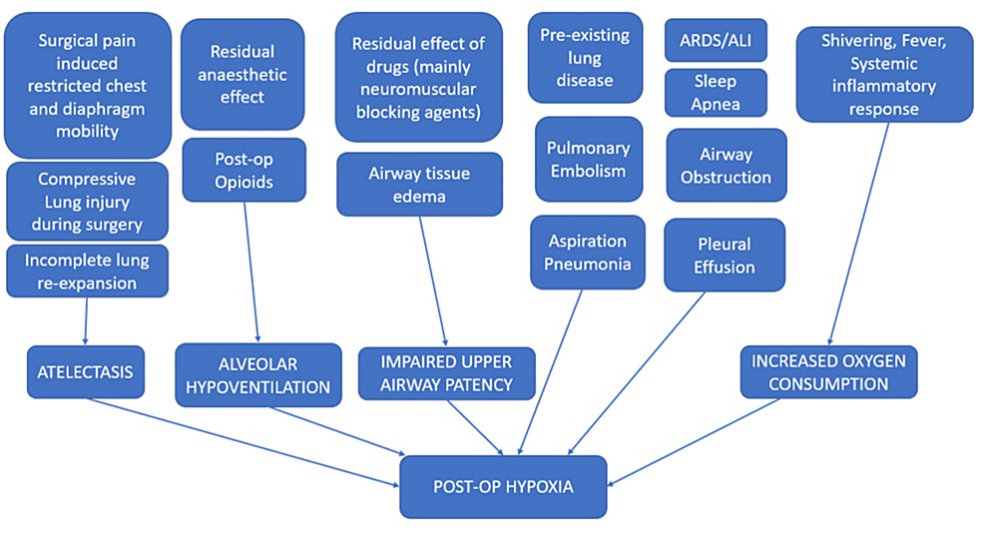
Possible mechanisms for postoperative hypoxia Figure credit: Authors; conceptualized from findings suggested by previously reported literature [12] Post-op: postoperative; ARDS: acute respiratory distress syndrome; ALI: acute lung injury

Postoperative hypoxia is a common challenge faced by surgeons. The earlier data suggested its incidence to be around 55% [9], but with the introduction of new and short-acting anesthetic drugs, minimally invasive surgical procedures, enhanced postoperative care, and the advent of day-care surgery, the incidence has fallen down to just around 20% in elective surgeries [10-11]. A catena of studies has highlighted the causes of postoperative hypoxia. These include but are not limited to incomplete lung re-expansion, pain-induced chest-wall/diaphragm mobility, prolonged surgery, a complication of pre-existing lung disease, residual effects of some drugs, and iatrogenic causes (Figure 7) [12].

Laparotomy is a major general surgical procedure that frequently proves to be life-saving. The existence of hemodynamic instability, prolonged surgical time, major surgical trauma, and associated mental trauma all push the major surgery of laparotomy to the verge of postoperative hypoxia. An interplay between the stated factors contributes to post-laparotomy hypoxia, forming it a less-reported identity, warranting attention.

Most cases present within a few hours of surgery with desaturation and are mainly attributed to prolonged drug effects [4]. Delayed presentation within days to week post-surgery is rarely seen these days and may be under-reported because of the dearth of data on post-laparotomy hypoxia.

In our case series, we reported five cases that developed post-laparotomy hypoxia, which was influenced by a myriad of factors as described in Table 1. The major cause that we could decipher was restricted diaphragmatic movement during and post-surgery. Incomplete lung base expansion during the long duration of laparotomy surgery, and later in the postoperative phase, the pain-induced restriction in diaphragmatic mobility highly contributes to postoperative hypoxia. Some specific causes were seen in individual cases: In Case 2 and Case 3, bronchoscopy revealed a mucus plug, which may have been the prime cause in those cases. In Case 4, a flare-up of *Pneumocystis carinii *infection was the main cause.

The findings in our case series were also in congruence with the findings of Melesse et al., who suggested longer duration of surgery had high chances of postoperative hypoxia [13]. The cases recovered well after being treated with supplemental oxygen and broad-spectrum antibiotics. The response to broad-spectrum antibiotics in some cases suggests a possible link between post-op infection and poor outcomes.

Perioperative physiotherapy has been shown to improve patient outcomes in abdominal surgeries. It can help prevent the number of PPCs [14]. In our cases, the patients were also promoted for incentive spirometry and were given chest physiotherapy post-surgery, which did contribute to a better recovery.

## Conclusions

Postoperative hypoxia presents a diagnostic challenge and requires timely suspicion and prompt intervention to eliminate the cause. We, therefore recommend the use of postoperative hypoxia oxygen support, peri-operative chest physiotherapy, and diligent monitoring of vitals in all laparotomy cases allowing efficient patient management. Future studies are warranted to explore the prevalence and possible causes of post-laparotomy hypoxia.
